# Latent virus infection upregulates CD40 expression facilitating enhanced autoimmunity in a model of multiple sclerosis

**DOI:** 10.1038/srep13995

**Published:** 2015-09-10

**Authors:** Costanza Casiraghi, Ana Citlali Márquez, Iryna Shanina, Marc Steven Horwitz

**Affiliations:** 1 Department of Microbiology and Immunology, The University of British Columbia, Vancouver, British Columbia, Canada, V6T 1Z3; 2 Present address: Department of Experimental, Diagnostic and Specialty Medicine, Alma Mater Studiorum-University of Bologna, Bologna, Italy

## Abstract

Epstein-Barr virus (EBV) has been identified as a putative environmental trigger of multiple sclerosis (MS) by multiple groups working worldwide. Previously, we reported that when experimental autoimmune encephalomyelitis (EAE) was induced in mice latently infected with murine γ-herpesvirus 68 (γHV-68), the murine homolog to EBV, a disease more reminiscent of MS developed. Specifically, MS-like lesions developed in the brain that included equal numbers of IFN-γ producing CD4^+^ and CD8^+^ T cells and demyelination, none of which is observed in MOG induced EAE. Herein, we demonstrate that this enhanced disease was dependent on the γHV-68 latent life cycle and was associated with STAT1 and CD40 upregulation on uninfected dendritic cells. Importantly, we also show that, during viral latency, the frequency of regulatory T cells is reduced via a CD40 dependent mechanism and this contributes towards a strong T helper 1 response that resolves in severe EAE disease pathology. Latent γ-herpesvirus infection established a long-lasting impact that enhances subsequent adaptive autoimmune responses.

Different autoimmune diseases have been linked to Epstein-Barr virus (EBV) infection: multiple sclerosis (MS), rheumatoid arthritis (RA), Sjogrens syndrome and systemic lupus erythematosus (SLE)^1^,^2^,^3^. Both MS and SLE patients have a dysregulated humoral and cellular immune response against EBV^4^,^5^,^6^,^7^,^8^,^9^,^10^,^11^. Cross-reactive antibodies and CD4^+^ T cells specific for both EBV proteins and self-antigen have been isolated from SLE and MS patients, respectively^10^,^12^. Epidemiological findings indicate that there is a tight correlation between EBV infection and development of these diseases. Particularly, mononucleosis, a disease caused by EBV, increases the risk of developing MS and SLE later in life^13^,^14^. Intriguingly and with controversy, EBV infection has been detected in both the central nervous system (CNS) and joints of MS and RA patients, respectively^1^,^15^,^16^,^17^. It was subsequently proposed that chronic EBV infection at the site of autoimmunity development (i.e. brain for MS and synovium for RA) could lead to initial inflammation that causes the first insult leading to the onset of autoimmunity^1^,^16^. Despite all of this evidence, it is still not clear how EBV would trigger autoimmunity.

Since EBV does not infect rodents, its murine homologue, murine γ-herpesvirus-68 (γHV-68) has been a biologically relevant tool in studying the interactions between the virus and host in mice^18^. γHV-68 elicits a similar immune response and pathology to EBV in mice that shares many features. Both viruses establish a life long infection in B cells, modulating the immune response of the host, leading to the expansion of a potent CD8 response similar to that detected in humans during mononucleosis^19^. Intriguingly, different studies have shown that some autoimmune diseases and chronic inflammation can be modulated by γHV-68 infection. Experimental autoimmune encephalomyelitis (EAE), a mouse model widely used to study MS in rodents, is exacerbated by acute γHV-68 infection^20^. More severe disease is also observed in γHV-68 infected mice in the context of inflammatory bowel disease^21^ and Crohn’s disease^22^. In all these studies, the mechanisms that the virus is exploiting to lead to these enhanced autoimmune phenotypes were not described. We previously demonstrated that EAE pathology is severely heightened in mice latently infected with γHV-68 and it is more reminiscent of MS including brain specific lesions with CD4^+^ and CD8^+^ T cells and demyelination^23^. Mice latently infected with γHV-68 were induced for EAE (γHV-68 EAE) and mounted a potent Th1 response that lacked an IL-17 response and leads to brain parenchyma inflammation. Whereas, in uninfected EAE mice, inflammation is confined in the spinal cords and it is driven by a mixed Th1-Th17 response. Antigen presenting cells (APCs) expressing higher levels of CD40 and MHC II were found to be responsible for the preferential Th1 skewing in γHV-68 EAE mice^23^. This suggests that a γ-herpesvirus is able to manipulate innate immunity and influence both the skewing and the strength of T cell responses upon a second pro-inflammatory stimulus. However, it remained to be addressed mechanistically how γHV-68 latency triggers the enhancement of the disease, specifically addressing whether the latent life cycle is required and what role upregulation of costimulatory molecules, in particular CD40, has in inciting enhanced EAE.

CD40 is a co-stimulatory molecule that is expressed on mature dendritic cells (DCs), B cells, monocytes, epithelial cells and endothelial cells^24^,^25^,^26^. The presence of microbial products or danger signals activates Toll-like receptors leading to the activation and maturation of DCs and, in particular, upregulation of surface expression of CD40. Type I interferons (Type I IFNs) play a critical role not only in the regulation of CD40 expression, but also in the control and maintenance of both γHV-68 acute and latent infection^27^. Type I IFNs serve to program DCs to drive Th1 responses through activation/phosphorylation of the STAT 1 pathway^28^,^29^. In addition to type I IFNs, the cognate interaction between CD40 and CD40 ligand (CD40L) enhances DC activation and it is important in providing T cell help to B cells, activating cytotoxic CD8^+^ T cells, directing a Th1 response and controlling regulatory T cells (Tregs)^24^,^30^,^31^. Interestingly, CD40 polymorphisms and dysregulation have been shown to play a role in the development of autoimmunity^32^.

We hypothesized that heightened CD40 expression was mechanistically participating in the development of severe EAE pathology. Our data demonstrate that CD40 is required for the efficient priming of strong Th1 responses and for decreased Tregs frequencies in mice latently infected with γHV-68. We were also able to show the converse using a latency deficient virus, in which we observed that enhancement of EAE pathology absolutely required γHV-68 latency. Essentially, the ability to skew the adaptive immune response towards a Th1 phenotype and retain low frequencies of Treg cells through CD40 expression and co-stimulation represents a novel mechanism in which latent γ-herpesviruses like EBV can act to facilitate and induce autoimmune disease.

## Results

### During acute infection with γHV-68, EAE is delayed

We have previously shown^23^ that mice latently infected with γHV-68 (five weeks post primary infection) present a heightened EAE pathology characterized by earlier onset of paralysis, more severe clinical symptoms, enhanced T cell infiltrations inside the CNS, and a potent Th1 response accompanied by downregulation of Th17 responses. Strikingly, γHV-68 EAE mice also showed CD8^+^ T cell infiltrations and myelin damage inside the brain parenchyma^23^, closely resembling the composition of immune infiltrates in MS plaques. Further, we observed an increase in surface expression of CD40 and MHC II on CD11b^+^ CD11c^+^ cells during the antigen presentation phase of EAE responsible for the enhanced Th1 responses detected in γHV-68 EAE mice^23^.

**Figure 1:**
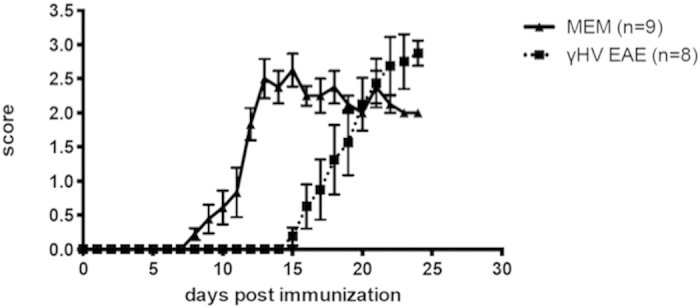
Acute γHV-68 infection delays the onset of EAE symptoms in C57Bl/6 mice. Mice were either infected with γHV-68 (γHV-68 EAE, dashed line) or MEM (MEM, solid line). Two days p.i., EAE was induced in both groups (day 0 on the graph). The graph shows clinical scores and numbers on the x-axis indicate days post EAE induction. Two separate experiments; data are represented as mean, error bars are SEM.

To determine if the establishment of latency by γHV-68 is a requirement in order to see enhancement of EAE symptoms, EAE was induced in mice during acute infection with γHV-68 before the latent life cycle was established. We observed that EAE was delayed during acute infection (Fig. 1) and the severity of the disease was similar between uninfected mice and γHV-68 acutely infected mice (Fig. 1). Interestingly, the onset of EAE symptoms in infected mice was concomitant with the date of establishment of γHV-68 latency (day 14/15) as was previously demonstrated by a number of different laboratories^33^,^34^,^35^.

### Mice infected with a latency deficient γHV-68 have a less severe EAE course and lower amounts of T cell infiltrations inside the CNS than mice infected with γHV-68

The observations that during acute infection, EAE onset was delayed and the disease scores were similar between uninfected mice and γHV-68 acutely infected mice suggested that establishment of latency prior to EAE induction is required for the development of enhanced disease. We hypothesized that the latency was indispensable for the enhancement of EAE symptoms, so we chose to study a recombinant γHV-68 (γHV-68 AC-RTA) in which the genes responsible for the establishment of latency have been deleted and the gene driving lytic infection is constitutively expressed^36^. In a manner similar to wild type virus, this recombinant virus acutely infects mice and stimulates a cellular and humoral immune response comparable to that elicited by the wild type virus^36^. As reported previously, no viral DNA was detected in the splenocytes of mice infected with this virus 15 days post infection^36^, indicating that latency was not established and that virus was cleared.

**Figure 2:**
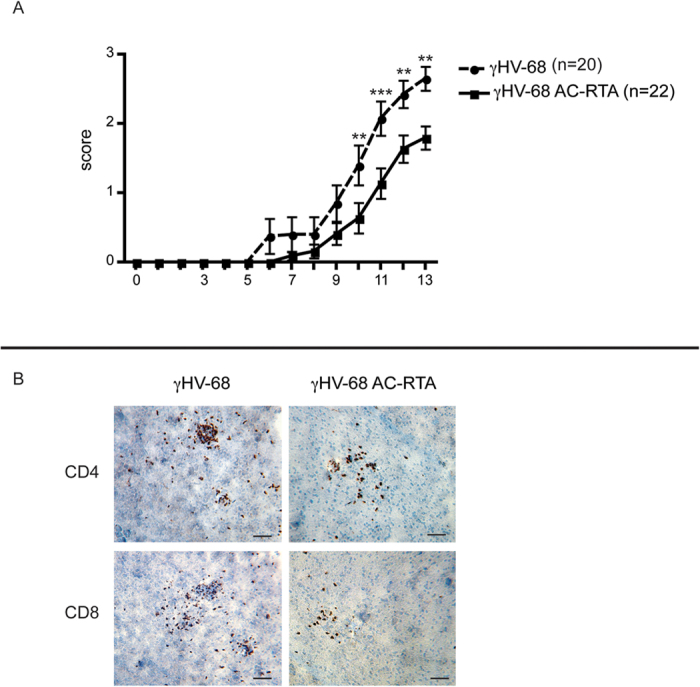
Mice infected with γHV-68 AC-RTA have a less severe EAE course and lower amounts of T cell infiltrations inside the CNS than mice infected with γHV-68. Mice were either infected with γHV-68 (γHV-68 EAE, dashed line) or γHV-68 AC-RTA (γHV-68 AC-RTA EAE, solid line). Five weeks p.i., EAE was induced in both groups (day 0 on the graph). **(A)** The graph shows clinical scores and numbers on the x-axis indicate days post EAE induction. **(B)** Two weeks post EAE induction, mice were perfused, brains were harvest, embedded in OCT, snap frozen, cut and stained with antibodies specific for CD4 and CD8. The images displayed are representative consecutive sections cut from the same cerebral hemisphere of the same mouse. Scale bar: 50 μm. Four separate experiments; data are represented as mean, error bars are SEM and were analyzed with two-way ANOVA followed by Bonferroni’s post-test: **p < 0.01; ***p < 0.001.

**Figure 3:**
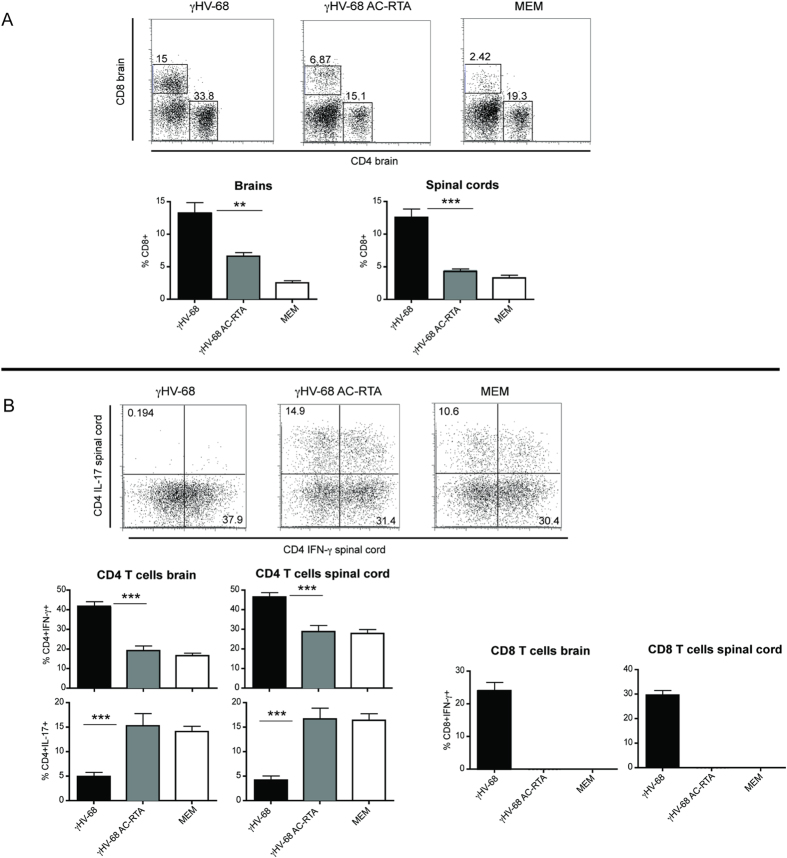
Mice infected with γHV-68 AC-RTA have very low CD8 T cell infiltrations and mount the same type of T helper response inside the CNS as naïve EAE mice. Mice were infected with γHV-68 (black bars) or γHV-68 AC-RTA (grey bars) or MEM only (open bars). Five weeks p.i. EAE was induced. Two weeks post EAE induction, mice were perfused, brain and spinal cords were harvested and immune infiltrations were isolated. Isolated immune cells were stimulated with PMA and ionomycin and stained for CD4 **(A)** and CD8 **(A)** to measure IFN-γ and IL-17 **(B)** production. The amount of CD8 T cells infiltrating inside the CNS of γHV-68 AC-RTA EAE and naïve EAE mice was too low to perform intracellular staining **(B)**. Three separate experiments with 5–6 mice/group; data are represented as mean, error bars are SEM and were analyzed with t-test: **p < 0.01; ***p < 0.001.

To directly determine the role of latency in this model, we characterized EAE development in γHV-68 AC-RTA mice. We assessed the differences in the immune cell composition inside the CNS and analyzed the T cell response after infection of wild type mice with either γHV-68 or γHV-68 AC-RTA followed by EAE induction. Similar to uninfected EAE mice, γHV-68 AC-RTA EAE mice showed milder clinical CNS symptoms (Fig. 2A), delayed disease onset (Fig. 2A) and fewer CD4^+^ and CD8^+^ infiltrations inside the brain parenchyma (Fig. 2B) when compared to γHV-68 EAE mice. In the end, the disease pathology of the γHV-68 AC-RTA EAE mice resembled those of uninfected EAE mice^23^. These data indicate that the heightened EAE pathology observed in mice latently infected with γHV-68 was not observed when mice were infected with a recombinant γHV-68 that was not able to establish latency. Further, γHV-68 AC-RTA EAE mice presented with reduced CD4^+^ and CD8^+^ T cell infiltrations in both the brains and the spinal cords when compared to γHV-68 EAE mice (Fig. 3A). Additionally they showed increased Th17 responses in the CNS (Fig. 3B) and a decrease in IFN-γ production in both CD4^+^ and CD8^+^ T cells. Overall, the type of immune response skewed by γHV-68 AC-RTA mice after EAE induction was more similar to naïve EAE mice than to γHV-68 EAE mice.

### Mice infected with the latency-free γHV-68 AC-RTA strain do not upregulate CD40 expression on antigen presenting cells upon EAE induction

**Figure 4:**
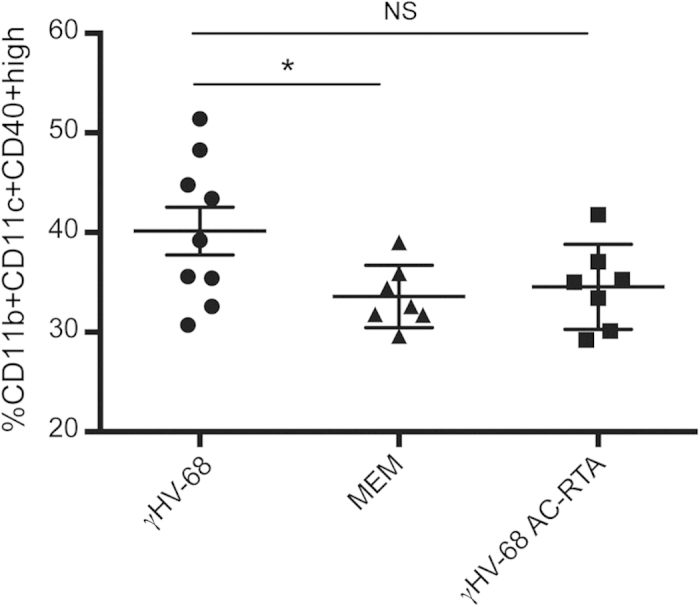
Infection with γHV-68 AC-RTA abolishes CD40 upregulation on antigen presenting cells upon EAE induction. Mice were infected with γHV-68 (black circles) or MEM only (black triangles) or γHV-68 AC-RTA (black squares). Five weeks p.i. EAE was induced. Four days post EAE induction, spleens were harvested and stained. Two separate experiments with 3–4 mice/group. All data are represented as mean, error bars are SEM and were analyzed with one way ANOVA comparing γHV-68 to MEM and γHV-68 AC-RTA: *p < 0.05.

As previously mentioned, we have already demonstrated that latently infected mice present with an upregulation of CD40 on APCs. We then sought to determine whether infection with γHV-68 AC-RTA would, in contrast to latent γHV-68 infection, not upregulate CD40 and also establish whether CD40 expression is an important key to immunological regulation during latent viral infections. APCs from C57Bl/6 mice infected with γHV-68 AC-RTA express CD40, during the antigen presentation phase of EAE at levels comparable to naïve EAE mice (Fig. 4) rather than the increased expression observed in γHV-68 latently infected mice (Fig. 4) thereby associating latent infection with increased CD40 surface expression and likely co-stimulation. From our prior manuscript, increased CD40 expression was defined on APCs identified by CD11b^+^, CD11c^+^ surface marker staining. This set of cells represents a broad spectrum of cell types and it is likely that only a small subset of APCs within this population has increased CD40 surface expression. The variable nature of this subset within the larger population is likely reflected in the variability of the mouse-to-mouse comparisons. Overall, the increase in CD40 surface expression is significantly greater from the cells isolated from γHV-68 latently infected mice.

### The presence of CD40 on antigen presenting cells is required to induce enhanced Th1 responses in mice latently infected with γHV-68

**Figure 5:**
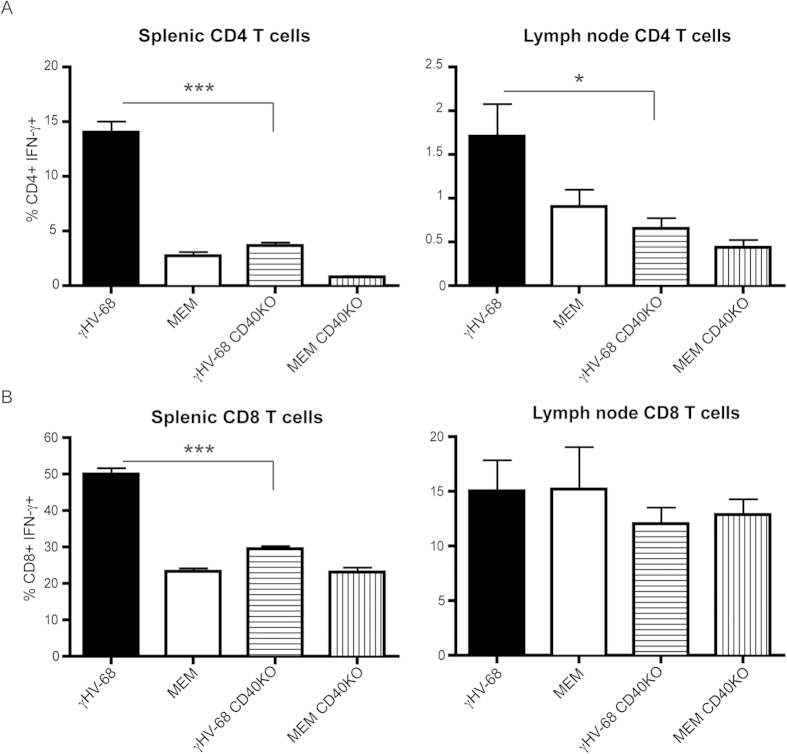
CD40 is required for enhanced Th1 responses in γHV-68 mice. C57Bl/6 wild type and C57Bl/6 CD40KO mice were infected with γHV-68 (black bars, horizontal lines bars) or MEM only (open bars, vertical lines bars). Five weeks p.i., spleens and inguinal lymph nodes were harvested. **(A,B)** Isolated immune cells were stimulated with PMA and ionomycin and stained for CD4 **(A)** and CD8 **(B)** to measure IFN-γ production. Three separate experiments with 3–5 mice/group; data are represented as mean, error bars are SEM and were analyzed with t-test (comparing γHV-68 with γHV-68 CD40KO): *p < 0.05; ***p < 0.001.

Enhanced Th1 responses and enhanced CD8^+^ T cell activation have been shown to be dependent on CD40^37^,^38^,^39^,^40^,^41^,^42^,^43^,^44^. Further, these characteristics were observed in γHV-68 EAE mice and in association with an upregulation of CD40^23^. Additionally, in γHV-68 AC-RTA EAE mice, brain inflammation is highly reduced, indicating that the priming of a strong Th1 response is required to drive both CD4^+^ and CD8^+^ T cells inside the brain parenchyma leading to the development of myelin lesion as observed in γHV-68 EAE mice^23^. To investigate the effect of the lack of CD40 during γHV-68 infection and latency, C57Bl/6 wild-type (wt) and C57Bl/6 CD40KO mice were infected or not with γHV-68. Five weeks post infection (p.i.), the level of IFN-γ produced by T cells was assessed. C57Bl/6 CD40KO mice infected with γHV-68 fail to mount the strong Th1 response typical of γHV-68 C57Bl/6 wt mice. In fact, IFN-γ production by CD4^+^ T cells in γHV-68 C57Bl/6 CD40KO mice was comparable to naïve wt mice both in the spleen and in the lymph nodes (Fig. 5A). IFN-γ produced by CD8^+^ T cells was reduced in the spleen of γHV-68 C57Bl/6 CD40KO mice when compared to γHV-68 C57Bl/6 wt. In contrast, there was no comparative reduction of IFN-γ in the lymph nodes (Fig. 5B).

**Figure 6:**
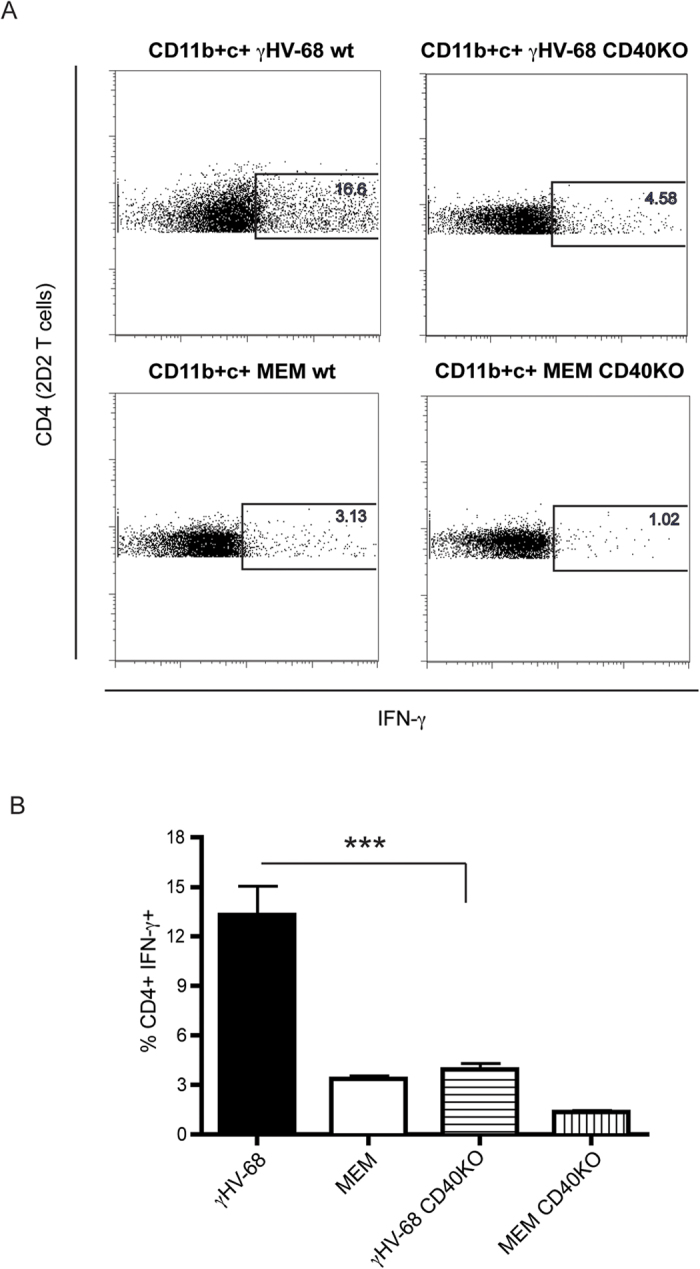
CD11b+ CD11c+ cells isolated from CD40KO γHV-68 EAE mice loose the ability to prime an enhanced IFN-γ response in 2D2 CD4+ T cells *in vitro*. C57Bl/6 wild type and C57Bl/6 CD40KO mice were infected with γHV-68 or MEM only. Five weeks p.i., EAE was induced. At day 4 post EAE induction, spleens and lymph nodes were harvested and CD11b + CD11c+ cells were isolated. CD4 T cells from 2D2 mice were isolated at the same time. CD11b + CD11c+ were incubated with 2D2 CD4 T cells and MOG peptide for 72 hours. T cells were restimulated and stained to assess the production of IFN-γ **(A)** Representative FACS plot showing IFN-γ production in 2D2 CD4 T cells after incubation with CD11b + CD11c+ cells isolated from a γHV-68 wt EAE mouse (upper left panel), or a γHV-68 CD40KO EAE mouse (upper right panel), or a naïve wt EAE mouse (lower left panel) or a naïve CD40KO EAE mouse (lower right panel). **(B)** Histogram showing the percentages of 2D2 CD4 T cells producing IFN-γ after incubation with CD11b+CD11c+ cells from a γHV-68 wt EAE mouse (black bar), or a naïve wt EAE mouse (open bar), or a γHV-68 CD40KO EAE mouse (horizontal lines bar), or a naïve CD40KO EAE mouse (vertical lines bar). Two separate experiments with triplicate wells for each group. Data are represented as mean, error bars are SEM and were analyzed with t-test (comparing γHV-68 with γHV-68 CD40KO): ***p < 0.001.

Additionally, as CD40 was found to be upregulated on the surface of CD11b^+^ CD11c^+^ cells upon EAE induction^23^, an *in vitro* antigen presentation assay was performed to assess if CD40 was critical to skew the potent Th1 response observed in γHV-68 EAE mice upon myelin peptide presentation. Transgenic T cells, bearing a TCR specific for myelin olygodendrocyte glycoprotein (MOG), were incubated with CD11b^+^ CD11c^+^ cells isolated from a γHV-68 EAE wt mouse and were primed. These cells produced significantly greater amounts of IFN-γ than when incubated with the same cell subset from uninfected EAE wt mice. This effect was abrogated if CD11b^+^ CD11c^+^ were taken from CD40KO mice (Fig. 6A,B). This result shows that CD40 expression on the surface of APCs is an absolute requirement to trigger enhanced IFN-γ production in T cells upon antigen presentation in mice latently infected with γHV-68.

### γHV-68 infection drives increased STAT1 responses in APCs

**Figure 7:**
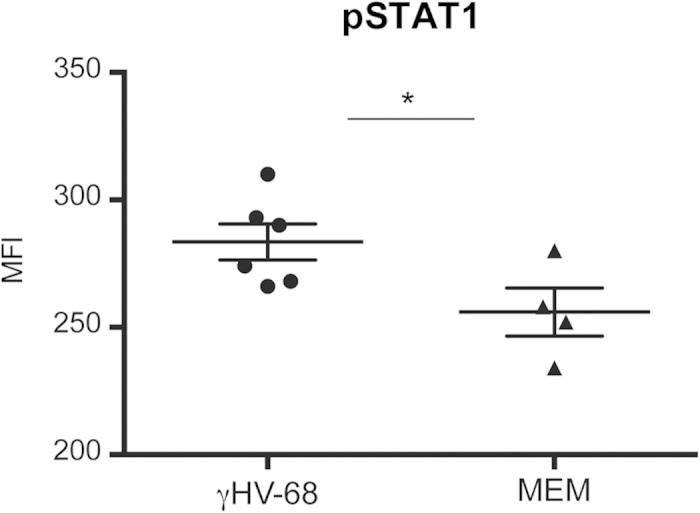
Latent infection with γHV-68 upregulates pSTAT1 levels on CD11b+CD11c+ cells. Mice were either infected with γHV-68 (black circles) or MEM only (black triangles). Five weeks p.i. spleens were harvested; CD11b + CD11c + cells were sorted and stained for pSTAT1. Graph shows MFI levels of pSTAT1 for each group. Two separate experiments with 2–3 mice/group. Data were analyzed with t-test: *p < 0.05.

Type I IFNs play a crucial role in the maintenance of latency of γHV-68^27^. In addition, upregulation of CD40 and MHCII on APCs is dependent on the presence of Type I IFNs^45^,^46^. Type I IFNs modulate APCs including dendritic cells to mediate Th1 responses by activating the STAT1 pathway^28^,^29^. We tried to measure the levels of Type I IFNs during γHV-68 latency with no success. The increase in Type I IFNs production during viral latency is likely localized to only a small subset of cells and reasonably difficult to measure. To indirectly measure the effect of IFN I, we chose to measure the levels of phosphorylation of STAT1 (pSTAT1). pSTAT1 is a signature molecule that defines the potential for Type I IFN mediated activity^28^,^29^. pSTAT1 can also be driven by other inflammatory mediators such as IFNγ, however by sampling during the inflammatory quiescent period prior to EAE, these alternative mediators may have a reduced contribution. More importantly, it is our premise that the APCs are programmed by the presence of a latent virus (in a different cell, memory B cell) and that this programming will lead to a greater phosphorylation of STAT1 and a greater STAT1 response. We harvested spleens from latently infected mice and sorted CD11b^+^ CD11c^+^ cells. We then determined pSTAT1 levels by flow cytometry. We observed that, even prior to EAE induction, pSTAT1 levels were increased in APCs harvested from γHV-68 latently infected mice (Fig. 7) as compared to those of uninfected mice. While these results suggest a potential link to IFN I, they leave open the possibility that other mediators are providing a greater STAT1 response. Overall, these results suggest that there is likely a strong association between γHV-68 induced upregulation of pSTAT1 levels and the upregulation of surface CD40 in APCs, ultimately driving the response towards a Th1 phenotype. Most importantly, it would be expected that a strong Th1 response would include both upregulation of pSTAT1 and CD40 in APCs.

### Mice latently infected with γHV-68 have decreased Treg in the periphery and in the CNS after EAE induction. Viral latency and induction of CD40 upregulation are required for this phenotype

**Figure 8:**
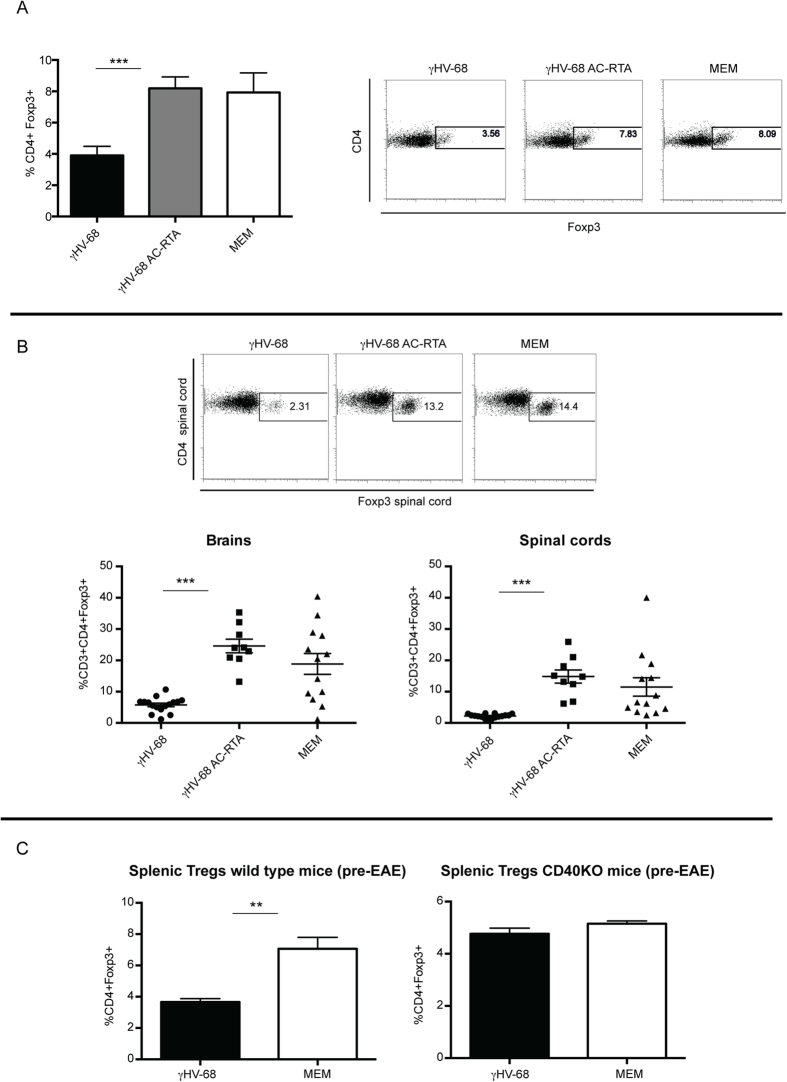
Regulatory T cells are decreased in spleens, brains and spinal cords of γHV-68 mice during EAE and this decrease is dependent on the establishment of latency and CD40 expression. Mice were infected with γHV-68 (black bars-black circles) or γHV-68 AC-RTA (grey bars-black squares) or MEM only (open bars-black triangles). Five weeks p.i. EAE was induced. Two weeks post EAE induction, **(A)** spleens were harvested and stained for Tregs. The right panels show representative FACS plots and the graph shows the percentage of Tregs (CD4 + Foxp3+). Three separate experiments with 3 mice/group. **(B)** Brain and spinal cords were harvested from perfused mice and immune infiltrations were isolated. Unstimulated immune cells were stained for CD3, CD4 and Foxp3 expression. Three separate experiments with 5–6 mice/group. **(C)** C57Bl/6 wild type and C57Bl/6 CD40KO mice were infected with γHV-68 (black bars) or MEM only (open bars). Five weeks p.i., spleens were harvested. Unstimulated splenocytes from wild type mice (left panel) and CD40KO mice (right panel) were stained for CD4 and Foxp3 expression. Three separate experiments with 3–5 mice/group. Data are represented as mean, error bars are SEM and were analyzed with t-test: ***p < 0.001, **p < 0.01.

In the periphery, Treg induction through APC:T cell costimulation, specifically CD40:CD40L, has been clearly demonstrated^38^,^41^. The increased surface expression of CD40 and potent Th1 response in γHV-68 infected mice led us to ask, if there were relevant changes in the frequency of Tregs. To specifically investigate the frequency of Tregs in the periphery and CNS we examined the Treg populations in γHV-68 latently infected mice and γHV-68 AC-RTA infected mice after EAE induction. The frequencies of Tregs were decreased in the spleens of γHV-68 EAE mice when compared to both γHV-68 AC-RTA EAE mice and naïve EAE mice (Fig. 8A). The same results were obtained when the frequencies of Tregs in the CNS were analyzed (Fig. 8B): the levels of Tregs in γHV-68 AC-RTA infected EAE mice were comparable to those of uninfected EAE mice. This suggests that viral latency likely through a CD40 mechanism has a role in controlling the frequencies of Tregs in γHV-68 mice. Finally, the frequency of Tregs was decreased in γHV-68 C57Bl/6 wt mice before EAE induction (50 days post γHV-68 infection), but it was rescued in γHV-68 C57Bl/6 CD40KO mice that showed the same Treg frequency as naïve CD40 KO mice (Fig. 8C). Since CD40 has been shown to be critical to control Treg frequencies^38^,^41^, it was expected that the Treg frequency on latently infected γHV-68 CD40KO mice would be similar to that observed in naïve uninfected CD40KO mice. Further, the lack of disease enhancement post EAE in mice infected with γHV-68 AC-RTA demonstrates that viral latency and its influence on innate immunity is critical to the control of Treg frequencies and adaptive immunity during latent γHV-68 infection.

## Discussion

Despite a large body of work that associates EBV infection to the development of autoimmunity, it is currently not clear what mechanisms this virus is exploiting to cause autoimmunity. Previous work focused primarily on EBV specific adaptive immunity in patients affected by autoimmunity and how this response is different when compared to healthy individuals. However the influence that EBV latency has on innate immunity has not been investigated in the context of autoimmune diseases. Previously, we used the murine equivalent to EBV, γHV-68, to demonstrate that γ-herpesviruses have the ability to modulate subsequent immune interactions and to specifically heighten EAE pathology to more resemble MS. Here, we determined that this enhanced disease requires γHV-68 latency and likely acts by upregulating CD40 surface expression on APCs. CD40 expression and co-stimulation is pivotal in controlling the type and strength of the adaptive immune response in response to a second pro-inflammatory stimulus such as EAE: CD40 co-stimulation and γHV-68 latency act to enhance both CD4^+^ and CD8^+^ effector T cell activation and reduce Tregs during EAE.

Specifically, we showed that γHV-68 enhanced T cell activation is accompanied by a decrease in Tregs frequencies both in the periphery and in the CNS during EAE. It has been previously shown that mice infected with γHV-68 present with decreased expression of Foxp3 and diminished T cell regulatory activity up to day 15 post γHV-68 infection^47^. Our results show for the first time, to our knowledge, that γHV-68 mice have decreased splenic percentages of Tregs and that this reduction is long lasting, still evident more than 50 days after infection. Further, this suppression of Treg frequency is removed in the absence of virus latency and CD40. We suggest that γHV-68 latency, in concert with the increase in CD40 expression on APCs, drives a decrease in Treg frequencies and thereby increases susceptibility to autoimmunity. Our data is supported by previous work that demonstrated that CD40 signaling suppresses the development of Tregs^38^,^41^. It is conceivable that decreased numbers of Tregs are contributing to the exacerbation of EAE symptoms observed in γHV-68 mice. In fact, it has been extensively shown that Tregs have an important role in preventing EAE development in mice (for a review^48^). Adoptive Treg transfers and treatment with monoclonal antibodies aimed at increasing the numbers of Tregs are both effective means to ameliorate EAE^48^. Interestingly, Tregs have decreased suppressive functions in MS patients^49^,^50^,^51^ and patients with mononucleosis have also decreased frequencies of Tregs in the blood^52^. A decrease in Tregs as a consequence of γ-herpesvirus infection could be a predisposing factor for the development of autoimmunity.

Additionally, it has been shown that CD40 is important for cytotoxic CD8^+^ T cell activation^37^,^40^,^42^,^43^, suggesting that increased CD40 expression in γHV-68 EAE mice may have a role in CD8^+^ T cell enhanced activation and CNS infiltration that we observed during EAE in latently infected mice. Our findings that enhanced CD8^+^ T cell activation during EAE is dependent on both CD40 expression and γHV-68 latency are in agreement with previous studies that have demonstrated that CD40L-CD40 interaction is required for the prolonged clonal expansion and activation of CD8^+^ T cells during the “mononucleosis like” phase of γHV-68 infection, coincident with latency establishment^53^. It has also been shown that enhancement of CD40 signaling substitutes for CD4^+^ T cell help during control of latency to prevent γHV-68 reactivation^54^. From these results, it is clear that CD40 is playing an important role especially during γHV-68 establishment of latency.

A question that remains open is how does γHV-68 control CD40 expression. We have previously shown that CD11c^+^ CD11b^+^ cells expressing high levels of CD40 are not infected by γHV-68. It is conceivable that the virus is using an indirect mechanism to upregulate CD40, possibly through Type I IFNs, however other mediators including IFNγ could be mechanistically important. Type I IFNs are produced during the infection and maintenance of γHV-68 latent infection^27^. γHV-68 latency requires type I IFN to maintain its latent life cycle^27^. Alternatively, a viral protein or components of the viral genome could bind to pattern recognition receptors and further activate APCs to produce increased amounts of co-stimulatory molecules like CD40. Intriguingly, MS patients upregulate CD40 in their peripheral blood^55^ and CD40 positive cells co-localize with T cells in active MS lesions^56^. Additionally interaction between CD40 and CD40L on T cells isolated from MS patients stimulates increased production of IL-12 by APCs^57^ and IFN-β, used as a therapeutic agent in MS, decreases the level of CD40L on T cells^58^.

The EBV gene product, LMP-1, is a decoy receptor for the CD40 receptor and can replace CD40 signaling in B cells^59^. This observation is particularly intriguing considering the tight link between EBV infection, mononucleosis and MS, the successful results of anti-B cell therapies in treating MS and the ability of B cells to act as APCs. While an LMP-1–like gene product has not yet been observed for γHV-68, the virus may influence CD40 signaling in a different manner because controlling co-stimulation is critical to EBV and γHV-68 pathogenesis. Our results suggest a possible, yet to be described, mechanism where EBV might be influencing autoimmunity in humans: EBV affects APCs leading to Th1 skewing, CD8^+^ T cell activation and decreased Tregs frequencies. MS and other autoimmune diseases such as Lupus in which EBV has been implicated are heterogeneous and it is conceivable that they do not have only one etiologic agent. It is likely that EBV could be the trigger of the disease in only a subset of patients. To address this, the activation status of APCs in humans affected by different sub-types of MS should be investigated and linked to both EBV infection/mononucleosis history and T helper responses/CD8^+^ activation status.

In conclusion, we have demonstrated that latent infection with γ-herpesviruses predisposes individuals to severe autoimmune disease by modulating DCs and suppressing Treg frequencies likely through DC modulation (CD40 expression) and this in turn, exaggerates CD4^+^ and CD8^+^ T cell aggression. This profound ability of γ-herpesviruses to influence autoimmunity represents a unique mechanism and offers a novel target for potential therapies.

## Materials and Methods

### Ethics Statement

All animal work was performed under strict accordance with the recommendations of the Canadian Council for Animal Care. The protocol was approved by the Animal Care Committee (ACC) of the University of British Columbia (certificate numbers: A0\415 and A08-0622).

### Infections and EAE induction

C57Bl/6 mice, C57Bl/6 CD40KO mice and 2D2 mice were purchased from the Jackson Laboratory and were bred and maintained in our rodent facility at the University of British Columbia. Mice were infected intraperitoneally (i.p.) between 7–10 weeks of age with 10^4^ pfu of γHV-68 WUMS strain (purchased from ATCC, propagated on BHK cells); or 10^4^ pfu of latency deficient γHV-68 AC-RTA (originally developed by Dr. Ting-Ting Wu, generous gift of Dr. Marcia A. Blackman)^36^; or 200 μl of MEM as a control. EAE was induced 35–40 days post infection by injecting subcutaneously each mouse with 100μl of emulsified complete Freund’s adjuvant (DIFCO) with 200μg of MOG 35-55 (GenWay biotech, purity >95%) and 400 μg of desiccated Mycobacterium tuberculosis H37ra (DIFCO). Mice also received two i.p. injections with 200 ng of pertussis toxin (List Biologicals) at time of immunization and 48 hours later. Mice were scored on a scale of 0 to 5: 0, no clinical signs; 0.5, partially limp tail; 1, paralyzed tail; 2 loss of coordinated movements; 2.5; one hind limb paralyzed; 3, both hinds limbs paralyzed; 3.5, hind limbs paralyzed, weakness in the forelimbs; 4, forelimbs paralyzed; 5, moribund or dead

### Immune cells isolation, staining and flow cytometry

Two weeks post EAE induction, mice were perfused with 30cc of PBS and spinal cords, brains, spleens, inguinal and cervical lymph nodes were isolated. A single cell suspension was generated from all the organs. Immune cells were further isolated from the CNS using a 30% Percoll gradient. For intracellular staining, CNS mononuclear cells, splenocytes and lymph nodes cells were stimulated for 4 hours in IMDM (Gibco) containing 10% FBS (Gibco), GolgiPlug (BD Biosciences), 10 ng/ml PMA and 500 ng/ml ionomycin. Antibodies for the cell surface markers were added to the cells in PBS with 2% FBS for 30 min on ice. After wash, cells were resuspended in Fix/Perm buffer (eBiosciences) for 30–45 min on ice, washed twice and incubated with Abs for intracellular antigens (cytokines and transcription factors) in Perm buffer (30 min, on ice). Fluorescently conjugated antibodies directed against CD11b (clone M1/70), CD11c (clone N418), CD4 (clone RM4–5), CD8 (clone 53-6.7), CD3 (clone eBio500A2), CD40 (clone 1C10), MHC II (clone M5), IL-17 (eBio17B7), IFN-γ (clone XMG1.2), Foxp3 (clone FJK-16s) were all purchased from eBiosciences. Samples were acquired using a FACS LSR II (BD Biosciences) and analyzed using FlowJo software (Tree Star, Inc.).

### Immunohistochemistry

Brains from perfused mice were frozen in OCT (Fisher Scientific) and ten-micron thick sections were processed for immunohistochemistry. Briefly, sections were fixed in ice cold 95% ethanol for 15 min and washed in PBS several times. This was followed by washes in TBS with 0.1% Tween (TBST) and incubation for 10 min with 3% H_2_O_2_ to block endogenous peroxidase. After washing, blocking buffer was added for 1 h (10% normal goat serum in PBS). Primary antibody was added overnight at 4 °C: purified rat anti-mouse CD4, anti-mouse CD8 and anti-mouse F4/80 (all from eBiosciences), diluted 1:100 in PBS 2% normal goat serum. After washes in TBST, the biotinylated secondary antibody (anti-rat IgG, mouse absorbed, Vector) was added for 1 h, diluted 1:200 in PBS 2% normal goat serum. After washes in TBST, the Vectastain ABC reagent was used (Vector) following manufacturer’s instruction. Then, DAB (Sigma) was added as a substrate and, after incubation for 8 min in the dark and several washes in distilled water, sections were counterstained with Harris hematoxylin for 20 seconds, in lithium carbonate for 30 sec, washed in several changes of distilled water and mount with VectaMount AQ (Vector).

### Antigen presentation assay *in vitro* and pSTAT1 staining of antigen presenting cells

Spleens and inguinal lymph nodes were harvested at day 4 post EAE induction. A single cell suspension was prepared and stained with anti-CD11c and anti-CD11b antibodies (see above for details). CD11c^+^ CD11b^+^ cells were sorted with a FACSAria cell sorter (BD Biosciences). CD4^+^ T cells from 2D2 mice were isolated from spleens with a CD4^+^ T cells negative selection kit following manufacturer’s instructions (STEMCELL technologies). Isolated CD11b^+^ CD11c^+^ (3–4 × 10^4^/well) and 2D2 CD4 T cells (5 × 10^5^/well) were seeded on a 24 well plate in RPMI, 10% FBS and Pen/Strep (all from GIBCO) with or without 100 μM MOG peptide. After 72 hours, T cells were restimulated with PMA, ionomycin and GolgiPlug and stained for CD4 and IFN-γ as described above.

For pSTAT1 staining: spleens were harvested 5 weeks post γHV-68 infection. CD11b^+^ CD11c^+^ cells were stained and sorted as above. Once sorted, cells were fixed with 4% paraformaldehyde and incubated for 10 min at 37 °C. Cells were then washed with 2% PBS/FBS and permeabilized with 90% methanol for 30 min on ice, and then washed and stained for pSTAT1 (clone BD 4a) for 1 hour at room temperature. Cells were acquired and analyzed as detailed above.

### Statistical analysis

Two-way ANOVA followed by Bonferroni’s post test was employed to compare EAE scores. Unpaired Student’s t-test or one way ANOVA was used for all the other analyses (GraphPad Prism).

## Additional Information

**How to cite this article**: Casiraghi, C. *et al.* Latent virus infection upregulates CD40 expression facilitating enhanced autoimmunity in a model of multiple sclerosis. *Sci. Rep.* **5**, 13995; doi: 10.1038/srep13995 (2015).
